# Chemoproteomic identification of molecular targets of antifungal prototypes, thiosemicarbazide and a camphene derivative of thiosemicarbazide, in *Paracoccidioides brasiliensis*

**DOI:** 10.1371/journal.pone.0201948

**Published:** 2018-08-27

**Authors:** Joyce Villa Verde Bastos Borba, Sinji Borges Ferreira Tauhata, Cecília Maria Alves de Oliveira, Monique Ferreira Marques, Alexandre Melo Bailão, Célia Maria de Almeida Soares, Maristela Pereira

**Affiliations:** 1 Laboratório de Biologia Molecular, Instituto de Ciências Biológicas, Universidade Federal de Goiás, Goiânia, Goiás, Brazil; 2 Laboratório de Produtos Naturais, Instituto de Química, Universidade Federal de Goiás, Goiânia, Goiás, Brazil; Louisiana State University, UNITED STATES

## Abstract

Paracoccidioidomycosis (PCM) is a neglected human systemic disease caused by species of the genus *Paracoccidioides*. The disease attacks the host’s lungs and may disseminate to many other organs. Treatment involves amphotericin B, sulfadiazine, trimethoprim-sulfamethoxazole, itraconazole, ketoconazole, or fluconazole. The treatment duration is usually long, from 6 months to 2 years, and many adverse effects may occur in relation to the treatment; co-morbidities and poor treatment adherence have been noted. Therefore, the discovery of more effective and less toxic drugs is needed. Thiosemicarbazide (TSC) and a camphene derivative of thiosemicarbazide (TSC-C) were able to inhibit *P*. *brasiliensis* growth at a low dosage and were not toxic to fibroblast cells. In order to investigate the mode of action of those compounds, we used a chemoproteomic approach to determine which fungal proteins were bound to each of these compounds. The compounds were able to inhibit the activities of the enzyme formamidase and interfered in *P*. *brasiliensis* dimorphism. In comparison with the transcriptomic and proteomic data previously obtained by our group, we determined that TSC and TSC-C were multitarget compounds that exerted effects on the electron-transport chain and cell cycle regulation, increased ROS formation, inhibited proteasomes and peptidases, modulated glycolysis, lipid, protein and carbohydrate metabolisms, and caused suppressed the mycelium to yeast transition.

## Introduction

Paracoccidioidomycosis (PCM) is chronic systemic mycosis in humans. The etiologic agents of PCM are fungi that belong to *Paracoccidioides* genus, which has five species with occurrences restricted to geographic areas from Argentina, Brazil, Peru, Paraguay and Venezuela: *Paracoccidioides brasiliensis*, *Paracoccidioides americana*, *Paracoccidioides venezuelensis*, *Paracoccidioides restrepiensis* (these four species belonged to the former *P*. *brasiliensis* species) and *Paracoccidioides lutzii* [1]. Although these different species cause the same diseases, *P*. *lutzii* presents many genetics differences when compared to the other species. Genomic comparisons revealed that *P*. *lutzii* genome is larger than other *Paracoccidioides* spp. and also encodes a larger number of genes [2]. Additionally, phylogenetic analysis showed the speciation event took place ~ 32 millions of years ago. Also, evidences show *P*. *lutzii* undergoes recombination independently of those in the other *Paracoccidioides* spp [3]. PCM is the main cause of death due to systemic mycosis in Brazil and was responsible for 51.2% of deaths in the period between 1996 and 2006. At temperatures below 26°C, these fungi grow n the mycelium form, whereas at the host temperature, they growth as yeasts. These species are saprobes in humid soils; inhalation of mycelium propagules and conidia reach the lungs and initiate the infection. Once in the lungs, the fungus can disseminate through the hematogenic or lymphatic systems to infect other organs such as the skin, mucosa, adrenal glands, bones, central nervous system, liver, and cardiovascular system [4,5].

The treatment of PCM depends on the disease severity. The currently used antifungals are amphotericin B, sulfadiazine, trimethoprim-sulfamethoxazole, itraconazole, ketoconazole, and fluconazole [6]. The light and moderate forms of the disease are most commonly treated with itraconazole or the combination of trimethoprim-sulfamethoxazole. In contrast, the severe forms are treated with amphotericin B [7]. These drugs are known to have many serious side effects; depending on the treatment length, they can cause comorbidities and may therefore not be completely effective owing to possible treatment non-compliance [8]. Hahn *et al*. [9] identified fungi that were resistant to the commonly used antifungal classes. Therefore, there is a need to find new therapeutic drugs that are more effective, less toxic, and result in fewer side effects. Our research group has invested efforts in finding new drug targets and antifungal candidates that meet those needs ([10–16]Freitas-Silva, in preparation).

Thiosemicarbazides (TSCs) are important molecules in organic synthesis, as they are easily modifiable and can undergo structural modifications to produce different compounds for many applications. TSCs are obtained by the reaction of isothiocyanates and hydrazines [17]; many TSC derivatives have shown biological effects, such as anticonvulsant [18], antimicrobial [19], antitumor [20], antituberculosis [21], antiparasitic [22], and antioxidant [23] activities. Monoterpenoids are also very biologically significant molecules, obtained from essentials oils of plants, which possess a wide range of bioactive properties. These components are suitable starting materials for organic synthesis as they can be largely produced as pure enantiomers and have key functional groups that can be chemically modified. In previous studies, the thiosemicarbazide derivative of camphene (TSC-C), prepared by the reaction between isothiocyanocamphene and hydrazine, inhibited the growth of the fungus *Trichophyton menthagophytes* through damage to the fungal cell wall [24]. Our group also demonstrated that TSC-C inhibited *P*. *lutzii* growth [16] and we attempted to understand how the compound affected fungal cells and induced cell death. For this purpose, we analyzed the proteome (Freitas-Silva, in preparation) and transcriptome of *P*. *lutzii* treated with TSC-C [16].

Chemoproteomics is a new technique that aims to identify the cellular targets of molecules through the demonstration of direct molecule-target interactions [25]. In this work, we performed chemoproteomic analysis to identify the proteins of *P*. *brasiliensis* that interacted with TSC and TSC-C. The results were compared with those of previous transcriptomic [16] and proteomic (Freitas-Silva, in preparation) studies of TSC-C inhibition of *P*. *lutzii*. Even though we are comparing different species, our data showed that they are responding similarly to the compounds, as we were able to recognize similar patterns of response among *P*. *brasiliensis* and *P*. *lutzii* to TSC and TSC-C. We also tested the enzymatic inhibition activities of some of the proteins bound to the compounds and evaluated the influence of TSC and TSC-C in the mycelium to yeast transition.

## Methods

### Source of TSC and synthesis of TSC-C

TSC is commercially available and it was purchased from Sigma Aldrich (Sigma-Aldrich, St. Louis, USA). The N(4)-[2,2-dimethyl-3-methylnorbornane]-thiosemicarbazide (TSC-C) was prepared as previously described [24].

### Culture conditions of *Pb*18

*P*. *brasiliensis* strain *Pb*18 (ATCC 3209, phylogenetic species S1) was used in this work. The fungus was cultivated on Fava-Netto agar medium (1.0% w/v peptone, 0.5% w/v yeast extract, 0.3% w/v protease-peptone, 0.5% w/v beef extract, 0.5% w/v NaCl, 4% w/v glucose, and 1.4% w/v agar, at pH 7.2)[26] at 36°C or 23°C for the growth of the yeast or mycelium phase, respectively. To conduct the experiments, the cells were transferred to a pre-inoculum in Fava-Netto liquid medium for 3 days.

### Minimal inhibitory concentration of TSC and TSC-C in *P*. *brasiliensis* yeast cells

Resazurin powder (Sigma Aldrich) was dissolved in sterile distilled water at final concentration of 0.02%, sterilized by filtration, and stored at 4°C until use. Two 500 μg/mL of TSC (5.47 mM) and TSC-C (2.2 mM) stock solutions were prepared and diluted to obtain the working concentrations of 250 μg/mL(TSC—2.7 mM, TSC-C– 1.1 mM), 125 μg/mL(TSC– 1,3 mM, TSC-C– 550 μM), 62.5 μg/mL (TSC—683 μM, TSC-C– 275 μM), 31.24 μg/mL (TSC-341 μM, TSC-C– 137 μM), 15.62 μg/mL (TSC– 170 μM, TSC-C– 69 μM), 7.81 μg/mL (TSC—85 μM, TSC-C– 34 μM), 3.9 μg/mL (TSC—42 μM, TSC-C– 17 μM), 1.9 μg/mL (TSC—21 μM, TSC-C– 8.6 μM).

The minimal inhibitory concentration **(**MIC) was determined in accordance with to the microdilution method described by the Clinical and Laboratory Standards Institute (CLSI). A total of 1×10^4^ cells/mL of *P*. *brasiliensis* yeast cells were inoculated per well in McVeigh and Morton (MMcM) liquid minimal medium supplemented with different concentrations of TSC. To determine the maximum growth rate (positive control), the control wells were incubated in culture medium in place of the 100 μL of test compound dilution. The plates were incubated at 36°C and shaken at 150 rpm for 48 h. To each well, 15 μL of resazurin solution was added, and the plate was returned to the incubator for 24 h. The IC_50_ was the concentration of the compound that resulted in 50% inhibition of fungal cell growth, based on absorbance measurements at 600 nm.

### Cytotoxicity of TSC and TSC-C

BALB/C 3T3 mouse fibroblasts were obtained from Banco de Células do Rio de Janeiro (BCRJ; Rio de Janeiro, RJ, Brazil) and cultured in Dulbecco’s Modified Eagle’s Medium (DMEM; Gibco, Grand Island, NY, USA) supplemented with New Born Calf Serum (NBCS). The assays were conducted in accordance with the official protocol of the Organization for Economic Co-operation and Development (OECD). Briefly, 3T3 cells were seeded into 96-well plates to form a sub confluent monolayer (1×10^4^ cells/well). The culture medium was removed and different concentrations of TSC and TSC-C (the same concentrations as in the MIC assays) were added to cells and the incubated for 48 h at 37°C in an atmosphere of 5% CO_2_. The controls cells were incubated with only medium. After the incubation period, the cells were washed once with PBS, neutral red dye was added, and the cells were incubated again for 3 h at 37°C in an atmosphere of 5% CO_2_. Subsequently, the cells were washed with PBS and neutral red desorb solution (50:1:49, v/v/v, ethanol: acetic acid: water) was added to the plates. After the plates were shaken for 20 min, the absorption at 540 nm was measured. The EC_50_ was the concentration that resulted in 50% inhibition of cell growth.

### Protein extract preparation

*Pb*18 yeast cells were grown for 3 days in Fava-Netto liquid medium at 36°C. Yeast cells were centrifuged (10,000 × *g*, 15 min, 4°C), and the soluble proteins were extracted by using extraction buffer (20 mM Tris-HCl pH 8.8, 2 mM CaCl_2_) with a protease inhibitor cocktail (GE Healthcare, Uppsala, Sweden). After the addition of glass beads (0.45 mm), the cells were lysed in a bead-beater and centrifuged again (10,000 x *g*, 15 min, 4°C). The protein concentration of the supernatant was determined by using the Bradford assay (Sigma-Aldrich).

### Compound immobilization and affinity purification

For the affinity chromatography assay, the AminoLink immobilization kit (Thermo Fisher Scientific, Walthan, USA) was used in accordance with the manufacturer’s instructions. Briefly, 2 mg of each compound was dissolved in 2 mL of coupling buffer (0.1 M sodium phosphate, 0.05% NaN_3_, pH 7.0), the resin was equilibrated to 25–28°C and 6 mL of coupling buffer was added and drained through the resin. Then, 2 mL of compound solution was added, and the resin was shaken for 1 h and 10 μL of 50 mM sodium cyanoborohydride (NaCNBH_3_) was added and shaken overnight at 4°C. The content was drained and the flow through was saved to calculate the coupling efficiency. The resin was washed with 4 mL of coupling buffer. In order to block the remaining active sites, 4 mL of quenching buffer (1 M Tris-HCl, 0.05% NaN_3_, pH 7.4) was drained through the resin, 40 μL of 50 mM NaCNBH_3_ was added and incubated for 30 min with agitation, and the resin content was drained. Then it was washed with 6 mL of washing buffer (PBS 10 mM, pH 7.4) and equilibrated in the same buffer.

Two mL of protein extract with a total protein content of 20 μg in the washing buffer was added and incubated for 1 h. The flow-through was collected and the resin was washed with 12 mL washing buffer. Then, 8 mL of elution buffer (0.1 M glycine-HCl, pH 2.5) was added, 1 mL of fractions was collected, and 50 μL neutralization buffer (1 M Tris, pH 9.0) was added to each fraction. The columns were regenerated by washing with 18 mL washing buffer and stored at 4°C with 8 mL washing buffer containing 0.05% sodium azide. Ten microliters of each eluted fraction were subjected to SDS-PAGE and the gel was silver stained. A control sample was obtained using a new resin and starting from the blockage step of the above protocol. This sample retrieved the unspecific interactions, which were removed from the final analysis.

### Sample preparation, nanoUPLC-MS^E^ acquisition, and protein classification

The eluted fractions were concentrated by using a 3 kDa molecular weight cut-off in an ultracel regenerated membrane (Millipore, Bedford, USA). The protein extract concentrations were determined by using the Bradford assay [27] and the extracts were prepared for analysis, as previously described [28], by using nano-scale ultra performance liquid chromatography combined with mass spectrometry with data-independent acquisitions (nanoUPLC-MS^E^). In this system, the trypsin-digested peptides were separated by using a nanoACQUITY UPLC (Waters Corporation, Milford, USA). The MS data obtained via nanoUPLC-MS^E^ were processed and examined by using the ProteinLynx Global Server (PLGS) version 2.5 (Waters). Protein identification analyses were performed as previously described [29]. The observed intensity measurements were normalized to the identified peptides of the digested internal standard, rabbit phosphorylase. Proteins that were present in the negative control and the samples were removed from the analysis. For protein identification, the *Paracoccidioides* genome database (http://www.broadinstitute.org/annotation/genome/paracoccidioides_brasiliensis/MultiHome.html) was used. Protein tables generated by PLGS were merged by using FBAT software [30], and the dynamic range of the experiment was computed by using MassPivot software. The identified proteins were classified according to the MIPS functional categorization (http://mips.helmholtz-muenchen.de/proj/funcatDB/) by using the online tool PEDANT (http://pedant.gsf.de/pedant3htmlview/pedant3view?Method=analysis&Db=p3_p28733_Par_brasi_Pb18).

### Formamidase enzymatic assay

First the protein extracts were incubated with different concentrations of TSC (1 mM, 125 μM, 32 μM and 15 μM) or TSC-C (1 mM, 62 μM, 18 μM and 9 μM).Then, formamidase activity was measured by monitoring the production of ammonia, as previously described [31]. Protein samples (500 ng) were added to 100 mM formamide, 100 mM phosphate buffer, pH 7.4, and 10 mM EDTA. The reaction mixture was incubated at 37°C for 30 min. Subsequently, 400 mL phenol-nitroprusside and 400 mL alkaline hypochlorite solution (Sigma Aldrich) were added. The samples were incubated at 50°C for 6 min and the enzymatic activity was monitored at 625 nm. The amount of ammonia released was determined through comparison with a standard curve. The specific activity was calculated as the measured absorbance divided by the amount of proteins in μg.

### Assay of mycelium to yeast transition

The transition from mycelium to yeast was performed in MMcM liquid minimal medium [32]. The cultivation temperature was changed from 26°C to 36°C to induce the mycelium to yeast transition. Prior to the temperature change, the mycelium cells were grown in liquid medium for 18 h at 26°C and treated with sub inhibitory concentrations of 85.3 μM TSC and 17.2 μM TSC-C. The cells were maintained at 36°C for 5 days and the appearance of yeast cells was observed each day by using a Neubauer chamber.

## Results and discussion

### Determination of the susceptibility of *P*. *brasiliensis* and mouse fibroblast BALB 3T3 cells to TSC and TSC-C

As TSC derivatives have shown many biological activities [18–20,23] and camphene is a monoterpene with established antimicrobial [33] and fungicidal activities [34], we synthesized the camphene derivative TSC-C, which was expected to demonstrate excellent biological potential. The results of the activity of TSC and TSC-C against *P*. *brasiliensis* yeast cells and the toxicity against mouse fibroblast BALB 3T3 cells are shown in Table 1. The MIC value for the inhibition of fungus growth was lower in TSC-C (79 μM)[16] than in TSC (344 μM).

**Table 1:** Inhibitory and cytotoxic effects of TSC and TSC-C to *P*. *brasiliensis* and fibroblast cells, respectively.

Compound	MIC (IC_50_)	Cytotoxicity (EC_50_)	Selectivity index
TSC	344 μM	> 5.4 mM	>15.69
TSC-C	79 μM	1.1 mM	13.92

Those results showed that the camphene N (4) substitution of TSC resulted in a cooperative effect of the biological activities of both TSC and camphene. The camphene group is a large nonpolar structure that appears to increase the stability of the compound and optimize interactions with proteins. TSC was less toxic to fibroblast cells than TSC-C. The EC_50_ of TSC was not reached at the highest concentration tested (5.4 mM), but the EC_50_ value for TSC-C was 1.1 mM. In both cases, the cytotoxic concentrations were higher than the IC_50_ concentrations.

Both compounds presented good selective toxicity indexes. As we could not find the concentration of TSC that kills 50% of fibroblast cells, the selectivity index was calculated based on the highest concentration tested, which was not sufficient to kill mouse cells. The selectivity index was 13.92 for TSC-C and ≥15.69 for TSC. Although TSC-C was more toxic than TSC, the amount of TSC-C needed to inhibit fungal growth was much lower than the toxic concentration. As TSC-C showed better inhibition of fungus growth and TSC showed lower cytotoxicity, we decided to continue the investigation of both TSC and TSC-C.

### Chemoproteomic determination of protein interacting to TSC and TSC-C

**Fig 1:**
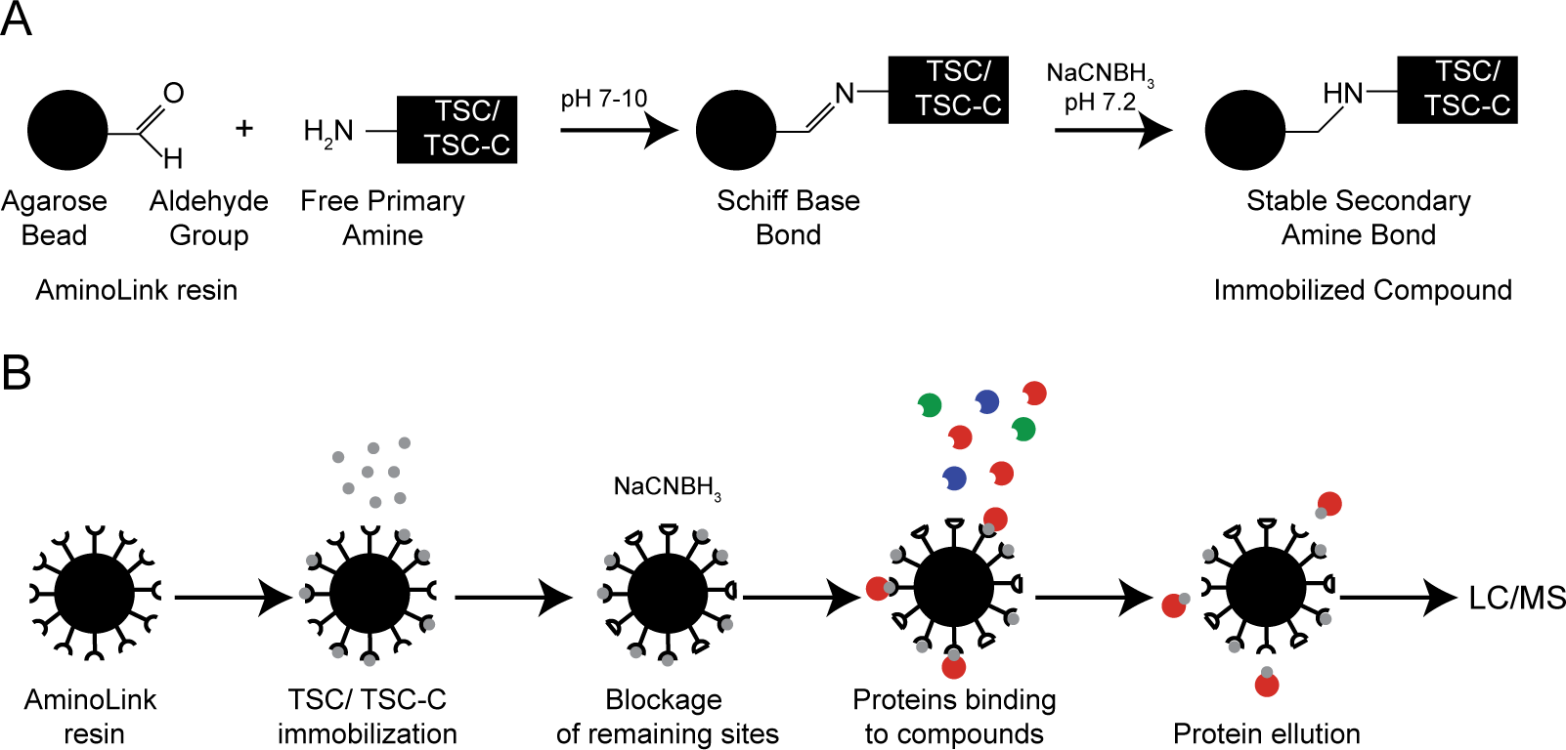
AminoLink resin protocol. ***Molecular view of the immobilization step (A) and overall view of the role protocol (B)*** AminoLink resins contain aldehyde groups that reacted with the free amino groups of TSC or TSC-C. The aldehyde sites that do not react with the compound are blocked with sodium cyanoborohydride (A). After compound immobilization, the fungal protein extract is added to the resin column. The proteins that interact with the compounds stay in the resin and the rest of the proteins are washed away. The interacting proteins are eluted from the resin, digested, and identified through mass spectrometry (B).

The compounds were immobilized in AminoLink (Thermo Fisher Scientific) resins through the free amino groups of the compound that reacted with the aldehyde groups of the resin to form Schiff base bonds that were stabilized by the addition of sodium cyanoborohydride, which forms a secondary stable amine bond (Fig 1). In order to evaluate the immobilization efficiency, the absorbance of TSC and TSC-C solutions was measured at 205 nm before and after the procedure. The absorbance of TSC prior to immobilization was 2.6 and decreased to 1.9 after resin immobilization; similarly, the absorbance of TSC-C decreased from 2.8 to 1.6. The decrease in absorbance demonstrated that part of the compounds in the solution was immobilized to the resin. The *P*. *brasiliensis* yeast soluble protein extracts were then incubated with the compounds on the immobilized resins. The proteins were eluted from the resins in eight fractions, each of 1 mL. As proteins were only detected in first two fractions from both resins, these fractions were submitted to proteomic analysis.

**Fig 2:**
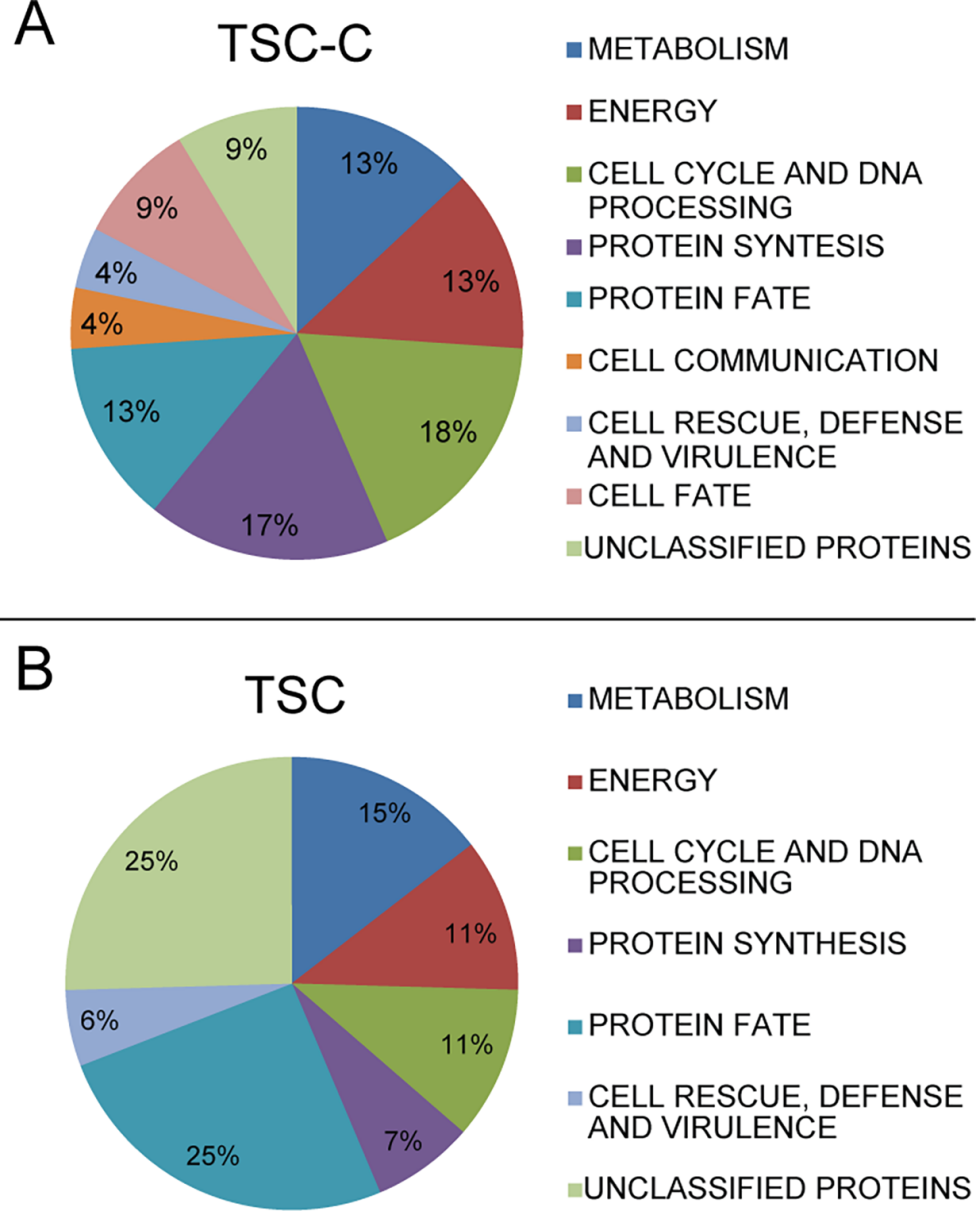
The graph indicates the statistically enriched MIPS functions. Proteins that were bound to TSC-C and TSC. The functional classification was based on the MIPS functional annotation scheme. Each functional class is represented as a color-coded segment and expressed as a percentage of the total number of proteins.

Proteomic analyses revealed that 23 proteins were bound to TSC-C and 55 proteins were bound to TSC (Table 2). The proteins eluted from the resin containing TSC-C were associated with metabolism (13%), protein synthesis (17%), energy (13%), cell rescue, defense, and virulence (4%), protein fate (13%), cell cycle and DNA processing (18%), cell fate (9%), cell communication (4%), and unclassified proteins (9%) (Fig 2A). Proteins eluted from the TSC resin were associated with metabolism (15%), energy (11%), protein synthesis (7%), protein fate (25%), unclassified proteins (25%), cell rescue, defense and virulence (6%), and cell cycle and DNA processing (11%) (Fig 2B). Some of these proteins were also identified in the proteome (Freitas-Silva, in preparation) and transcriptome [16] of *P*. *lutzii* growth in the presence of TSC-C.

**Table 2:** Functional classification of *P*. *brasiliensis* proteins interacting to TSC-C and TSC.

Accession number/ Protein classification	Protein description	TSC-C	TSC
**METABOLISM**	** **		
**Amino acid metabolism**			
PADG_06252	1,2-Dihydroxy-3-keto-5-methylthiopentene dioxygenase		**x**
PADG_01621	Aspartate aminotransferase		x
PADG_00663	Homoserine dehydrogenase		x
PADG_01314	YggS family pyridoxal phosphate enzyme		x
PADG_06546	Puromycin sensitive aminopeptidase		x
**Nitrogen, sulfur and selenium metabolism**			
PADG_06490	Formamidase	x	x
PADG_00331	Uricase		x
**C-compound and carbohydrate metabolism**			
PADG_02271	Alcohol dehydrogenase 1^*a*^	x	
PADG_07615	Glucan-1,3-β-glucosidase		x
**Lipid, fatty acid and isoprenoid metabolism**			
PADG_01291	Enoyl CoA hydratase isomerase ^*b*^	x	
**ENERGY**			
**Glycolysis and gluconeogenesis**			
PADG_05109	2,3-bisphosphoglycerate independent phosphoglycerate mutase		**x**
PADG_11132	Phosphoglucomutase		**x**
**Pentose-phosphate pathway**			
PADG_04604	Transketolase		
**Electron transport and membrane-associated energy conservation**			
PADG_01440	ADP ATP carrier protein	x	
PADG_03747	Alternative oxidase	x	
PADG_08024	Cytochrome c1	x	
PADG_06196	NADPH dehydrogenase		x
PADG_05523	Quinone oxidoreductase		x
PADG_07836	Quinone oxidoreductase		x
**CELL CYCLE AND DNA PROCESSING**			
**DNA processing**			
PADG_04144	ATP dependent RNA helicase eIF4A	x	
PADG_05798	Single stranded DNA binding protein	x	
PADG_05676	Ataxia telangiectasia mutated		x
PADG_03905	Proliferating cell nuclear antigen		x
**Cell Cycle**			
PADG_07248	Carboxy terminal kinesin 2	x	
PADG_02763	Cyclin dependent kinase regulatory subunit		x
PADG_03073	Nuclear movement protein nudC		x
PADG_05615	Ran specific GTPase activating protein		x
**RNA synthesis/processing**			
PADG_02659	Nucleoside diphosphate sugar epimerase	x	
PADG_05393	mRNA decapping hydrolase		x
**PROTEIN SYNTESIS**			
**Translation**			
PADG_02056	50S ribosomal protein L7/L12	x	
PADG_01220	60S ribosomal protein L13	x	
PADG_04449	60S ribosomal protein L23	x	x
PADG_05264	Ribosomal protein L19	x	
PADG_07863	40S ribosomal protein S8		x
PADG_02142	60S ribosomal protein L5		x
PADG_04588	60S ribosomal protein L22		x
**PROTEIN FATE**			
**Protein folding and stabilization**			
PADG_07599	Peptidyl-prolyl cis-trans isomerase ^*a*^ ^*b*^	x	x
PADG_04092	Peptidyl-prolyl cis-trans isomerase B		x
PADG_05203	Peptidyl-prolyl cis-trans isomerase ssp 1		x
PADG_11110	Golgi apparatus membrane protein TVP18		x
**Protein/peptide degradation**			
PADG_03221	Thimet oligopeptidase	x	x
PADG_06290	Proteasome component PRE5	x	
PADG_03982	Proteasome component C1		x
PADG_03967	Proteasome component C5		x
PADG_03680	Proteasome component PRE2		x
PADG_02735	Proteasome component PRE6		x
PADG_03727	Proteasome component PUP1		x
PADG_04067	Proteasome component PUP3		x
PADG_07190	Proteasome component Y7		x
PADG_00615	Proteasome component C7		x
PADG_07422	Serine proteinase		x
**CELL COMMUNICATION**			
**Celullar signalling**			
PADG_08337	GTP binding protein rhoA	x	
**CELL RESCUE, DEFENSE AND VIRULENCE**			
**Stress response**			
PAAG_05142	10 kDa heat shock protein mitochondrial	x	
PADG_04984	Hsp 10		x
PADG_02845	Diploid state maintenance protein chpA		x
PADG_02981	ThiJ/Pfpl family protein		x
**CELL FATE**			
**Cell death**			
PADG_06941	Mitochondrial fission 1 protein	x	
**Cell Wall**			
PADG_06336	Cell lysis protein cwl1	x	
**UNCLASSIFIED PROTEINS**			
PADG_08715	Hypothetical protein	x	
PADG_03273	Hypothetical protein	x	
PADG_04636	Dienelactone hydrolase family protein		x
PADG_08034	Dienelactone hydrolase family protein		x
PADG_05356	Isochorismatase domain containing protein		x
PADG_05798	Hypothetical protein		x
PADG_02343	Hypothetical protein		x
PADG_02764	Hypothetical protein		x
PADG_00183	Hypothetical protein		x
PADG_01867	Hypothetical protein		x
PADG_04057	Hypothetical protein		x
PADG_01220	Hypothetical protein		x
PADG_05239	Hypothetical protein		x
PADG_00921	Hypothetical protein		x
PADG_04439	Hypothetical protein		x
PADG_08116	Hypothetical protein		x

^*a*^ Regulated on transcriptome

^*b*^ Regulated on proteome

### Influence of TSC and TSC-C on enzymatic activity of formamidase

In order to evaluate if the compounds inhibited the enzymatic activity of interacting proteins, the activity of formamidase was tested. A crude protein extract of *P*. *brasiliensis* was obtained and then incubated with either TSC or TSC-C at 1 mg/mL. Then, the activity of formamidase was measured. Formamidase was found to interact to TSC and TSC-C,. The inhibition of formamidase by TSC and TSC-C was dose-dependent (Table 3).

**Table 3:** Inhibitory effect of TSC and TSC-C in the activities of formamidase.

	TSC (1 mM)	TSC (125 μM)	TSC (32 μM)	TSC (15 μM)	Control
**Specific activity:**	3.5 ± 0.28	24.11 ± 4.04	50.31 ± 3.42	74.55 ± 5.79	87.25 ± 8.81
	**TSC-C (1 mM)**	**TSC-C (62 μM)**	**TSC-C (18 μM)**	**TSC-C (9 μM)**	
**Specific activity:**	3.67 ± 0.29	39.99 ± 5.55	78.20 ± 5.32	124.98 ± 8.50	

Both compounds were able to interact with formamidase, an enzyme that converts formamide into formate and ammonia. The enzyme has a function in nitrogen metabolism and may be involved in host tissue damage or resistance to acidic environments. Formamidase was found on the cytoplasm and cell wall of *P*. *brasiliensis*. The enzyme was established to be an antigenic factor because it was reactive with the patient’s serum and may be a virulence factor [35]. Formamidase was up regulated in the transcriptome of yeast recovered from infected mice, in the transcriptome fungus yeast phase upon incubation with human plasma in comparison with human blood [36], and in the mycelia secretome [37]. Formamidase was down regulated in the proteome of *Paracoccidioides* spp. in zinc deprivation conditions [38]. Zinc is a very important micronutrient as it is a required cofactor for many enzymes and transcription factors [39]; consequently, zinc deprivation is a common host defense mechanism used by macrophages to inhibit the growth of pathogens [40]. Secreted proteins are important for the survival of pathogens in the host environment. These proteins can be related to many important functions, such as the provision of nutrients, cell-to-cell communication, detoxification of the environment, and removal of potential competitors [41]. During infection, the fungus must adapt its metabolism to survive in the host environment. Many genes contribute to *P*. *brasiliensis* adaption and survival in the host’s milieu during infection [42]. The interaction of TSC and TSC-C with formamidase was confirmed as the compounds inhibited formamidase activity. As formamidase is important for fungal pathogenesis, the interaction of the compounds with this protein may contribute to the abrogation of the fungal defenses to the host stressor agents, which would decrease the survival of the pathogen.

### Influence of TSC and TSC-C in the mycelium to yeast transition

**Fig 3:**
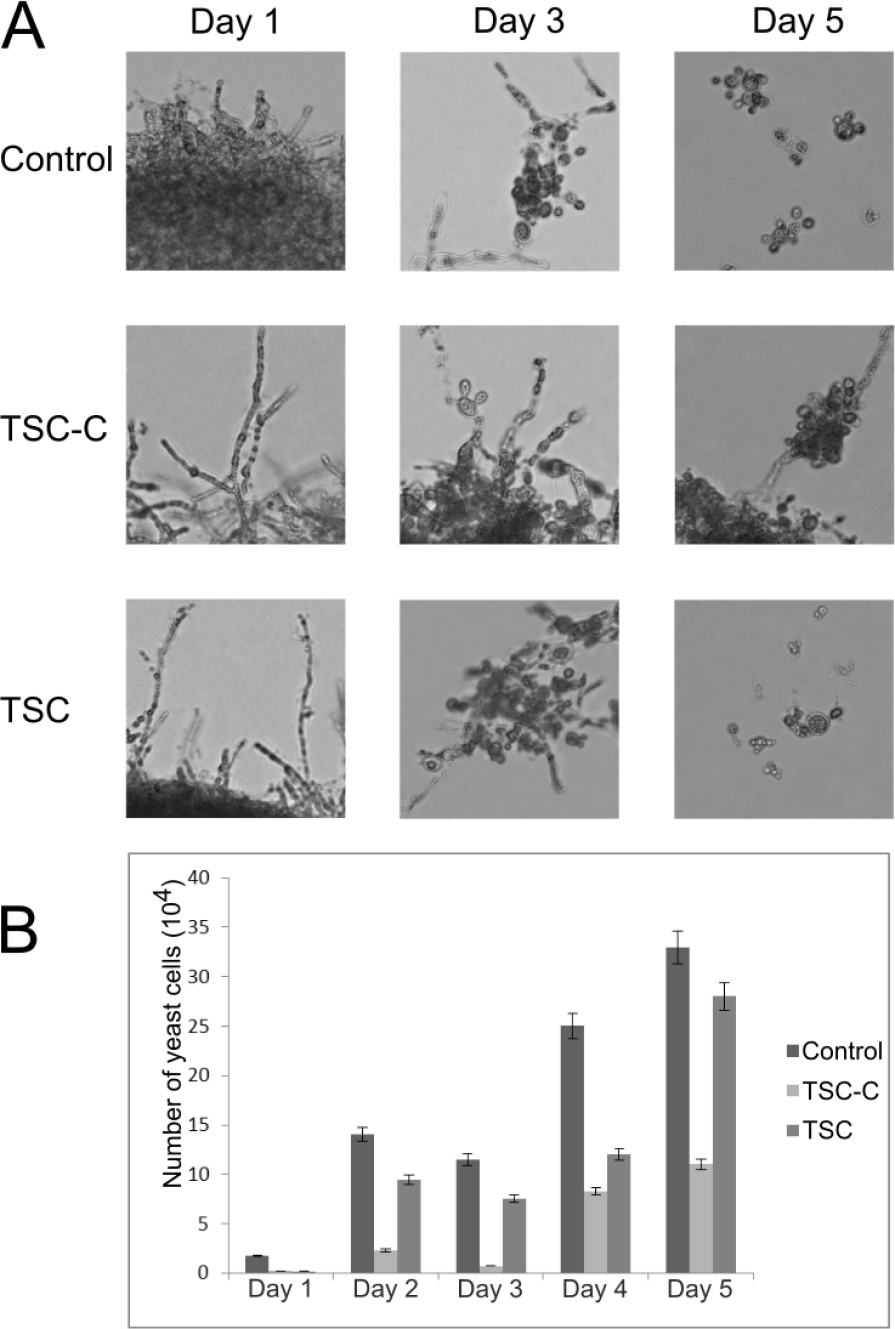
Effects of TSC and TSC-C on *P*. *brasiliensis* transition from mycelium to yeast. Mycelium cells were incubated for 5 days at 36°C on medium containing 85.3 μM TSC or 17.2 μM TSC-C. Cell morphology was observed by optical microscopy **(A)**. Yeast cells were counted by using a Neubauer chamber **(B)**.

*P*. *brasiliensis* is usually found in the soil as a saprobes mycelium and propagules that, when inhaled, may infect the host and differentiate into yeast cells in the host’s lungs. The transition from mycelium to yeast is very important in the development of infection [43]. As the interaction of TSC and/or TSC-C to proteins regulated during dimorphic transition, such as phosphoglucomutase, transketolase, formamidase, and aspartate aminotransferase [44–46], we performed a transition assay in the presence of TSC and TSC-C to investigate if these compounds were able to inhibit the transition of the fungus. The dimorphic transition from mycelium to yeast was monitored over 5 days. The fungus at 1, 3, and 5 days after transition is shown in Fig 3A; the results demonstrated that TSC-C strongly inhibited the morphological transition. TSC slowed the transition and influenced yeast morphology, resulting in smaller yeast cells in comparison with the control (Fig 3A). After 5 days, the percentage of cells that transitioned from mycelium to yeast in comparison with the control cells was 33.3% and 84.8% in the presence of TSC-C and TSC, respectively (Fig 3B).

The structure of the cell wall is subject to constant change, for example, during cell expansion and division, spore germination, and the mycelium to yeast transition. The enzyme β-1,3-glucosidase is very important to the cell wall modification processes because it is involved in β-glucan mobilization [47,48]. As TSC interacts with this enzyme, it could interfere with cell wall remodeling and thereby affect the mycelium to yeast transition. The arrest of the cell cycle in the G1 phase in the presence of TSC-C [16] may be another reason for the interference of the compounds in the dimorphic transition.

Moreover, as nucleotide-diphospho-sugars are the building blocks of various glycoconjugates and polysaccharides, they are implicated in the synthesis of different cell wall polysaccharides in plants [49]. The nucleoside diphosphate sugar epimerase is one of the nucleotide diphospho-sugar interconversion enzymes. The interconversion enzymes can synthesize many different polysaccharides from fructose 6-phosphate or from alternative pathways, such as the salvage pathway, or by simply recycling free sugars from cell wall degradation [50]. TSC-C bound to nucleoside diphosphate sugar epimerase, which might contribute to the impairment of the mycelium to yeast transition, because this process involves extreme cell wall remodeling.

### Main cellular processes influenced by TSC and TSC-C based on transcriptomics, proteomics, and chemoproteomics

Our findings highlighted TSC and TSC-C as multitarget compounds, as they could interact with many different targets in different pathways. Recently, multitarget drugs have increased in popularity owing to their potential synergistic effects, evasion of biological system adaptations that result from compensatory systems and redundant functions, and also avoidance of resistance linked to disease[51,52]. Furthermore, based on proteomic (Freitas-Silva, in preparation) and transcriptomic data [16], TSC and TSC-C result in a “butterfly effect”, in which a perturbation at one point in the system leads to larger perturbations in other points and, ultimately, to a massive global effect [53].

Transcriptomic data [16] revealed mitochondrial membrane disruption: after treatment with TSC-C, *P*. *lutzii* lost membrane mitochondrial potential, which prevented ATP synthesis. Transcripts such as NADH-ubiquinone oxidoreductase, NADH iron-sulfur dehydrogenase, cytochrome c oxidase chain VII, and ATP synthase D were affected after 8 h exposure to TSC-C. Proteomic data (Freitas-Silva, in preparation) showed that TSC-C interfered with energy production. The expression of 27 energy-related proteins was altered after 12 h incubation with TSC-C. Among the energy-related proteins, ATP synthase subunit beta, two ATP synthase gamma subunits, F-type ATPase subunit H, V-type ATPase subunit G, and vacuolar ATP synthase subunit E were down regulated; conversely, the mitochondrial F1F0 ATP synthase subunit and vacuolar ATP synthase subunit B were up regulated. The chemoproteomic data showed that TSC-C bound to the ADP-ATP carrier protein, alternative oxidase, and cytochrome c1, whereas TSC bound to NADPH dehydrogenase and to two quinone oxidoreductases. Collectively, the data indicated that the mitochondrial electron transport membrane was one of the pathways targeted by TSC and TSC-C. Transcriptomic analysis [16] also revealed increased ROS production and the up regulation of superoxide dismutase, an antioxidant enzyme. However, this could be a consequence of mitochondrial membrane disruption, as ATP synthase inhibitors such as oligomycin and apoptolidin have been shown to trigger ROS production [54,55].

Another ATP synthase inhibitor, citreovidin, was tested against malignant breast cancer cells and was found to inhibit cell proliferation. Proteomic analysis of the malignant cells indicated that citreovidin induced cell cycle arrest through the activation of the unfolded protein response by proteasomal proteins, such as 26S proteasome. When bortezomib, a 26S proteasome inhibitor, was tested in combination with citreovidin, non-apoptotic cell death occurred [56]. TSC-C bound cell cycle related protein carboxy terminal kinesin 2 and proteasome component PRE, whereas TSC bound cyclin dependent kinase regulatory subunit, nuclear movement protein nudC, ran-specific GTPase-activating protein, and eight proteasome component proteins. Transcriptomic data [16] indicated that the fungus experienced cell cycle arrest after the up regulation of proteins related to the cell cycle, such as nuclear segregation protein Bfr1, cell division control protein, subunit of condensin complex, and nuclear movement protein nudC, which occurred after TSC-C treatment; furthermore, the cell cycle arrest was confirmed experimentally. Proteomic data (Freitas-Silva, in preparation) further endorsed cell cycle arrest as several proteins were altered by exposure to the compound, including haloacid dehalogenase (HAD) superfamily hydrolase, cell division control protein 11, subunit of condensin complex, NEDD8 activating enzyme E1 regulatory subunit, DNA damage checkpoint protein RAD24, deubiquitination protection protein DPH1, ran-specific GTPase-activating protein, phosphopantothenoylcysteine decarboxylase, Arp2/3 complex subunit, and mitogen activated protein kinase MKC1. The 26S proteasome proteins were regulated in the proteome of TSC-C-treated cells and proteolytic activity was inhibited by TSC-C (Freitas-Silva, in preparation), which suggested that TSC-C might act in a similar manner to citreovidin with respect to the unfolded protein response and cell cycle arrest.

TSC and TSC-C were bound to the protein thimet oligopeptidase, a metallopepidase that hydrolyzes bioactive peptides such as encephalin, luliberin, and bradykinin [57]. Bradykinin, which is involved in inflammation, triggers the production of IL12 by dendritic cells that lead to the T helper 1 response. The hydrolysis of bradykinin by thimet oligopeptidase may modulate the immune response of the host and ease parasite evasion [58]. Compound interactions may inhibit this protein, which might stop fungal invasion of the host’s immune system.

The protein family peptidyl prolyl cis-trans isomerases (PPI) are enzymes that catalyze the cis-trans isomerization of peptidyl-prolyl peptide bonds. Their first characterized function was related to protein folding and chaperoning, but evidence of their participation in many other cellular processes has accumulated. Fungal PPIs are known to respond to stress tolerance, pathogenicity/virulence, protein folding, protein trafficking, immune functions, and cell cycle regulation [59]. In cell cycle regulation, bacterial PPIs can regulate cell division through the modulation of the functions of various other proteins that are indirectly related to the process [60]. TSC and TSC-C bound PPIs; proteins from this family were up regulated in the transcriptome [16] and down regulated in the proteome (Freitas-Silva, in preparation). Given the many processes in which these proteins are involved, targeting their inhibition may be an effective strategy. Considering their transcript and protein modulation, these proteins are likely to be very important to the fungus.

Many metabolic pathways are influenced by TSC and TSC-C. The proteomic analysis (Freitas-Silva, in preparation) indicated the down regulation of glycolysis and glyconeogenesis, most probably owing to the disruption of ATP synthesis. The transcriptome [16] showed that alcohol dehydrogenase was down regulated by four-fold and TSC-C was found to interact with this protein. Carbohydrate metabolism was also down regulated in the proteome (Freitas-Silva, in preparation); specific emphasis should be given to isocitrate dehydrogenase, an enzyme from glyoxylate cycle, a pathway responsible of generating intermediate glucose precursors. In addition, enzymes such as mannosyl transferase and mannitol-1-phosphate-5-dehydrogenase were down regulated in the transcriptome [16] and proteome (Freitas-Silva, in preparation), respectively. The lack of intermediate glucose precursors and the repression of some cell wall carbohydrate remodeling enzymes might contribute to deficiencies in the mycelium to yeast transition.

With regard to lipid metabolism, the proteins related to fatty acid synthesis were up regulated in the transcriptome [16] and proteome (Freitas-Silva, in preparation). Proteins related to beta-oxidation, such as enoyl-CoA hydratase and 3-ketoacyl CoA thiolase were down regulated at the protein level (Freitas-Silva, in preparation). TSC-C also bound to enoyl-CoA hydratase, which might be another target of the compound. The up regulation of fatty acid synthesis and the downregulation of beta-oxidation might be related to the need to renovate lipids damaged by TSC-C treatment.

The expression of several proteins associated with amino acid metabolism was altered and two proteins from this category were induced in the transcriptome [16]. The fungus is probably trying to adapt its metabolism to produce or degrade intermediate compounds that can be used in other pathways, such as energy production or the synthesis of structural macromolecules.

**Fig 4:**
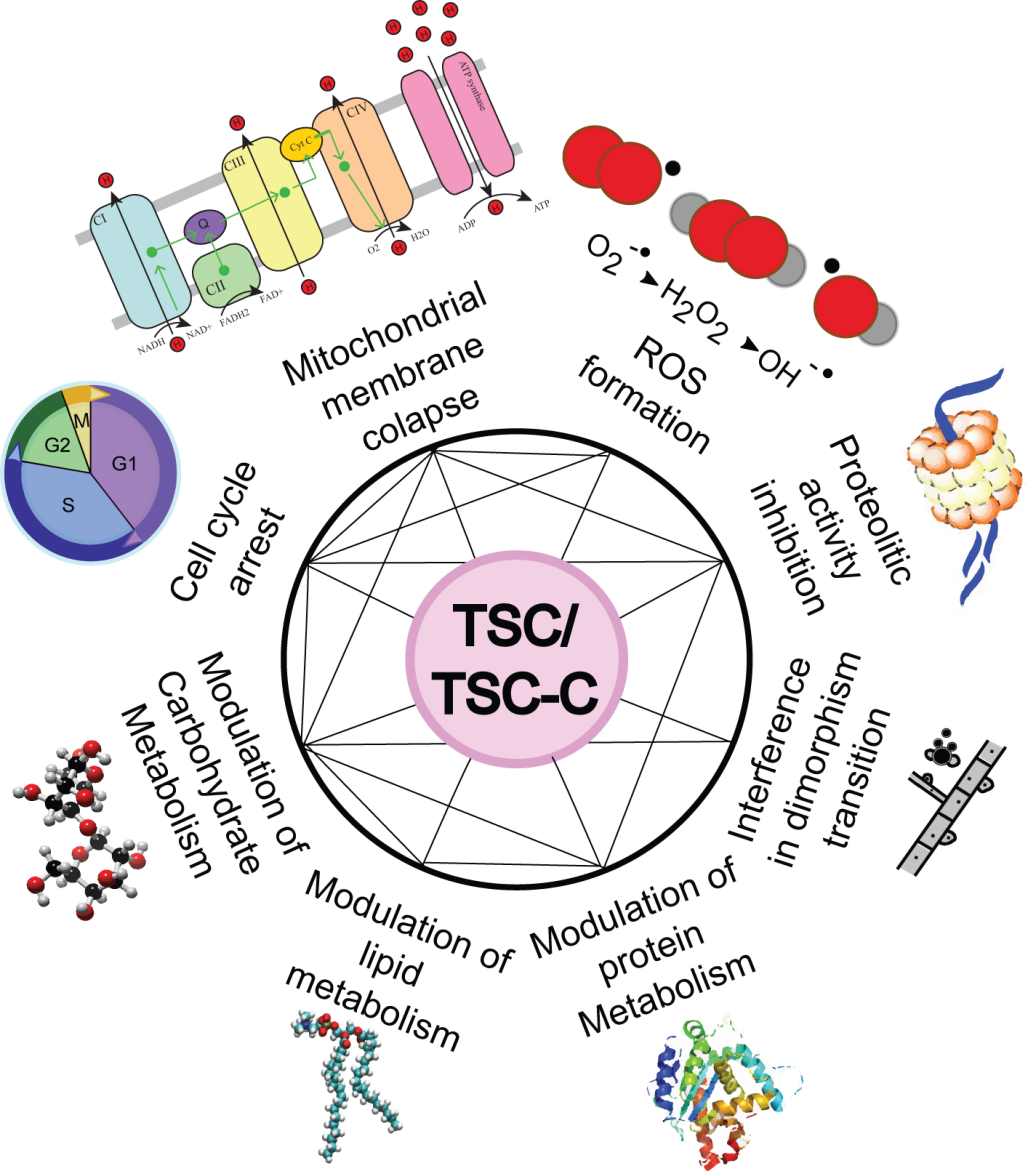
Main cellular processes influenced by TSC and TSC-C based on a comparison between the transcriptomic, proteomic, and chemoproteomic data.

In short, we think that TSC and TSC-C might be multitarget compounds that affected the electron-transport chain and cell cycle regulation, increased ROS formation, inhibited proteasomes and peptidases, modulated glycolysis, carbohydrate metabolism, and lipid and protein metabolism, and caused a deficiency in the mycelium to yeast transition (Fig 4).

## Conclusion

TSC and TSC-C were effective antifungals prototypes because they inhibited the growth of *P*. *brasiliensis* and were not cytotoxic to fibroblast cells at their IC_50_ concentrations. Through chemoproteomic analysis and comparisons of the transcriptomic and proteomic changes in fungal growth in the presence of these compounds, we were able to propose a robust mode of action for TSC and TSC-C (Fig 4). TSC and TSC-C act on the electron transport chain, preventing energy accumulation, and leading to oxidative stress. The compounds also exerted effects on cell cycle regulation, protein modulation, lipid and carbohydrate metabolism, interfered in cell transition, and altered fungal morphogenesis.
